# Anti-MOG antibodies are present in a subgroup of patients with a neuromyelitis optica phenotype

**DOI:** 10.1186/s12974-015-0256-1

**Published:** 2015-03-08

**Authors:** Anne-Katrin Pröbstel, Gabrielle Rudolf, Klaus Dornmair, Nicolas Collongues, Jean-Baptiste Chanson, Nicholas SR Sanderson, Raija LP Lindberg, Ludwig Kappos, Jérôme de Seze, Tobias Derfuss

**Affiliations:** Department of Neurology, University Hospital Basel, Petersgraben 4, 4031 Basel, Switzerland; Department of Biomedicine, University of Basel, Hebelstrasse 20, 4031 Basel, Switzerland; Department of Neurology, Hôpital de Hautepierre, University Hospital Strasbourg, 1 Avenue Molière, 67100 Strasbourg, France; Institute of Clinical Neuroimmunology, University Hospital Grosshadern, Max-Lebsche-Platz 31, 81377 Munich, Germany

**Keywords:** Neuromyelitis optica, Neuromyelitis optica spectrum disorder, Anti-aquaporin-4 antibodies, Anti-MOG antibodies, Inflammatory demyelinating CNS disease

## Abstract

**Background:**

Antibodies against myelin oligodendrocyte glycoprotein (MOG) have been identified in a subgroup of pediatric patients with inflammatory demyelinating disease of the central nervous system (CNS) and in some patients with neuromyelitis optica spectrum disorder (NMOSD). The aim of this study was to examine the frequency, clinical features, and long-term disease course of patients with anti-MOG antibodies in a European cohort of NMO/NMOSD.

**Findings:**

Sera from 48 patients with NMO/NMOSD and 48 patients with relapsing-remitting multiple sclerosis (RR-MS) were tested for anti-aquaporin-4 (AQP4) and anti-MOG antibodies with a cell-based assay. Anti-MOG antibodies were found in 4/17 patients with AQP4-seronegative NMO/NMOSD, but in none of the AQP4-seropositive NMO/NMOSD (*n* = 31) or RR-MS patients (*n* = 48). MOG-seropositive patients tended towards younger disease onset with a higher percentage of patients with pediatric (<18 years) disease onset (MOG+, AQP4+, MOG−/AQP4−: 2/4, 3/31, 0/13). MOG-seropositive patients presented more often with positive oligoclonal bands (OCBs) (3/3, 5/29, 1/13) and brain magnetic resonance imaging (MRI) lesions during disease course (2/4, 5/31, 1/13). Notably, the mean time to the second attack affecting a different CNS region was longer in the anti-MOG antibody-positive group (11.3, 3.2, 3.4 years).

**Conclusions:**

MOG-seropositive patients show a diverse clinical phenotype with clinical features resembling both NMO (attacks mainly confined to the spinal cord and optic nerves) and MS with an opticospinal presentation (positive OCBs, brain lesions). Anti-MOG antibodies can serve as a diagnostic and maybe prognostic tool in patients with an AQP4-seronegative NMO phenotype and should be tested in those patients.

## Findings

### Introduction

Neuromyelitis optica (NMO) is a clinically defined entity within the spectrum of inflammatory demyelinating diseases of the central nervous system (CNS) which is characterized by inflammatory attacks that are confined to the spinal cord and the optic nerves [1,2]. Limited forms of the disease are considered as NMO spectrum disorder (NMOSD) [3]. The finding of anti-aquaporin-4 (AQP4) antibodies in the majority of patients with NMO [4] and some patients with NMOSD has advanced our pathogenic understanding of the disease [5] and has directed the therapeutic approach towards a B cell-directed therapy [6]. However, 10% to 50% of NMO patients, depending on cohorts and assays used, are AQP4-negative [7]. Recent evidence suggests that some of the NMO cases are related to antibodies against myelin oligodendrocyte glycoprotein (MOG) [8-17].

Previously, we showed that anti-MOG antibodies are present in about 25% of pediatric patients with a first episode of acute demyelination and that these antibodies correlate with the disease course [18,19]. The aims of the present study were a) to analyze the presence of anti-MOG antibodies in an independent blinded cohort of patients with NMO/NMOSD and multiple sclerosis (MS) using the previously described cell-based assay (CBA) [18], b) to correlate antibody findings to clinical and magnetic resonance imaging (MRI) parameters of MOG-seropositive and AQP4-seropositive NMO patients and NMO patients with no detectable antibodies, and c) to characterize the long-term clinical outcome of the MOG-seropositive patients.

### Methods

A total of 135 patients including patients with NMO/NMOSD (*n* = 48), relapsing-remitting MS (*n* = 48), and healthy donors (*n* = 39) were analyzed. NMO/NMOSD and MS patient samples were collected at the University Hospital, Strasbourg, France between 2006 and 2012. The clinical data were obtained retrospectively from the European Database for Multiple Sclerosis (EDMUS). Healthy donor samples were obtained from the blood donation center, Etablissement Français du Sang (EFS), Strasbourg, France. Diagnoses of NMO/NMOSD or MS were based on the revised Wingerchuk criteria or the McDonald criteria, respectively [2,20]. Baseline sera for the NMO and MS patients were collected within an average of 8 years (0 to 42 years) (MOG vs. AQP4 vs. seronegative: 17 (3 to 32), 6 (0 to 42), 7 (0 to 15) years) and 14 years (3 to 37 years) of the first inflammatory episode, respectively. The mean period of observation for the NMO/NMOSD patients was 19 years (3 to 35) for the MOG-positive patients, 11 years (3 to 44) for the AQP4-positive patients, and 9 years (2 to 17) for the seronegative patients. Anti-AQP4 antibodies were measured by two different methods: indirect immunofluorescence (iIF) and CBA. Anti-MOG antibodies in the sera were measured by flow cytometry using a CBA with full-length, human, native conformational MOG as previously described [18]. The analysis was carried out blinded. Anti-MOG antibody positivity was determined by the ratio of the geometric mean channel fluorescence (GMCF) of the MOG-transfected and the empty vector-transfected cell line. The cutoff was calculated to be 1.45 (mean GMCF ratio plus two standard deviations of the healthy donor control group measured in parallel). The study was approved by the local ethical committee of the University Hospital of Strasbourg (DC-2009-1002; CPP 09/40; DC-2014-2222), and all patients gave their informed consent for the study.

### Results

**Figure 1:**
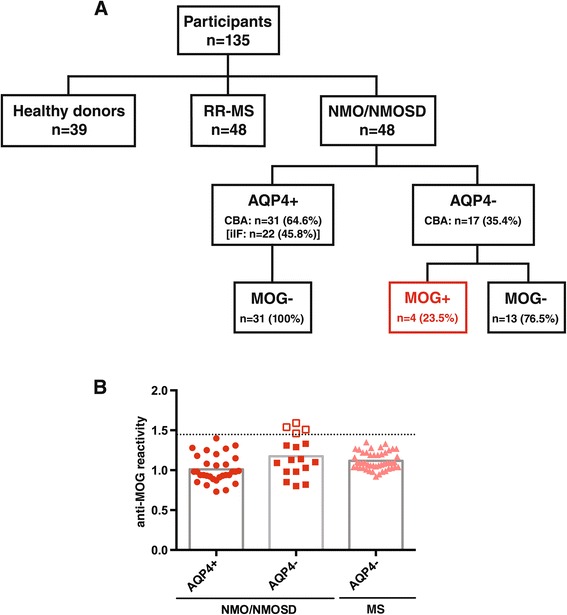
**Overview of patient cohort, antibody status, and anti-MOG antibody levels. (A)** Patient cohort and antibody status: The study comprised a total of 135 participants including patients with NMO/NMOSD (*n* = 48), relapsing-remitting multiple sclerosis (RR-MS) (*n* = 48), and healthy donors (*n* = 39). Of the NMO/NMOSD patients, 31 were positive for anti-AQP4 antibodies (AQP4+) with the cell-based assay (CBA) while only 22 patients tested positive with indirect immunofluorescence (iIF). All of the AQP4-seropositive patients were negative for anti-MOG antibodies (MOG−). Of the AQP4-seronegative patients (AQP4−), four patients had anti-MOG antibodies (MOG+) (CBA), while 13 patients had no detectable antibodies against either AQP4 or MOG. **(B)** Anti-MOG antibody levels: Anti-MOG reactivity was analyzed with the CBA and is expressed as the geometric mean channel fluorescence (GMCF) ratio of the MOG-transfected cell line divided by the empty vector-transfected cell line. Values shown represent the (mean) GMCF ratio of one to four experiments. The cutoff used (dotted line) is the mean GMCF ratio of the healthy donor group measured in parallel (*n* = 39) plus two standard deviations (cutoff = 1.45). Using this cutoff, 4 of the 17 (23.5%) NMO/NMOSD sera were positive for anti-MOG antibodies (empty red squares), all of which were AQP4-seronegative (filled red squares). None of the AQP4-seropositive NMO/NMOSD patients (filled red circles) and none of the RR-MS patients (filled light red triangles) were positive for anti-MOG antibodies.

In total, 48 patients with NMO/NMOSD were included in the study: 4 of the 48 patients tested positive for anti-MOG antibodies, all of which were negative for anti-AQP4-antibodies (Figure 1). Tested with the iIF assay, 22 patients were positive for anti-AQP4-antibodies, while testing with the CBA revealed that 31 patients were anti-AQP4 antibody-positive (Figure 1A). Only one patient was tested positive with the iIF assay, but negative with the CBA. The remaining 13 patients were seronegative for both antibodies tested (Figure 1).

The median age at first attack was slightly younger in the MOG-seropositive group (31 years; range 15 to 55 years) compared to the age of the AQP4-seropositive group (38 years; range 15 to 60 years) or the seronegative group (36 years; range 23 to 53 years). Of note, two out of four of the MOG-seropositive patients had a disease onset in childhood (<18 years), whereas this was the case only in three out of thirty one of the AQP4-seropositive and none of the seronegative patients. In terms of clinical presentation, half of the MOG-seropositive (2/4) or AQP4-seropositive (16/31) patients and about two thirds (8/13) of the seronegative patients had an initial presentation with uni-/bilateral optic neuritis (ON) (Table 1). A quarter of the MOG-seropositive (1/4) and about a third of the AQP4-seropositive (10/31) and seronegative (4/13) patients had a disease manifestation with transverse myelitis (TM). Remarkably, the mean time to the second attack affecting a different CNS region was longer in the MOG-seropositive patients (11.3 years; range 1 to 30 years) as compared to the AQP4-seropositive (3.2 years; range 0 to 9 years) and seronegative (3.4 years; range 0 to 7 years) patients (Table 1). Oligoclonal bands (OCBs) were more often found in the MOG-seropositive patients (3/3, not available in 1) compared to the AQP4-seropositive (5/29, not available in 2) and seronegative (1/13) patients.

**Table 1:** **Clinical, paraclinical, and MRI characteristics of MOG-seropositive NMO patients compared to AQP4-seropositive and AQP4/MOG-seronegative NMO/NMOSD patients**

**Characteristics**	**MOG+ (** ***n*** **= 4)**	**AQP4+ (** ***n*** **= 31)**	**MOG−/AQP4− (** ***n*** **= 13)**
Sex, female	3/4	23/31	8/13
Age (years), median (range)			
At first attack	31 (15 to 55)	38 (15 to 60)	36 (23 to 53)
At sampling	48 (47 to 56)	46 (20 to 72)	44 (23 to 66)
Age at first attack <18 years	2/4	3/31	0/13
Clinical presentation at first attack			
ON (uni-/bilateral)	2/4	16/31	8/13
TM (%LETM)	1/4 (100)	10/31 (90)	4/13 (100)
Both	1/4	5/31	1/13
Second territorial involvement			
ON (uni-/bilateral)	1/4	9/31	4/13
TM (%LETM)	2/4 (100)	13/31 (92)	8/13 (100)
NMOSD	0/4	4/31	0/13
None	1/4	5/31	1/13
Time to second territory involvement (years), mean (range)^c^	11.3 (1 to 30)	3.2 (0 to 9)	3.4 (0 to 7)^a^
EDSS, mean (range)			
At sampling	3.6 (2.5 to 5.0)	3.0 (0 to 8.0)	3.5 (0 to 6.5)
At last follow-up	3.6 (2.5 to 5.0)	3.1 (0 to 10.0)	3.9 (0 to 10)
CSF, OCB pos.	3/3^a^	5/29^b^	1/13
MRI brain lesions	2/4	5/31	1/13
Immunomodulatory treatment	4/4	27/31	12/13
Clinical follow-up (years), mean (range)	19 (3 to 35)	11 (3 to 44)	9 (2 to 17)

^a^Not available *n* = 1; ^b^Not available *n* = 2; ^c^Excluding patients with combined ON/TM at disease onset. Abbreviations: AQP4, aquaporin-4; CSF, cerebrospinal fluid; EDSS, Expanded Disability Status Scale; LETM, longitudinally extensive transverse myelitis; MOG, myelin oligodendrocyte glycoprotein; MRI, magnetic resonance imaging; NMO, neuromyelitis optica; NMOSD, neuromyelitis optica spectrum disorder; OCB, oligoclonal bands; ON, optic neuritis; TM, transverse myelitis.

**Figure 2:**
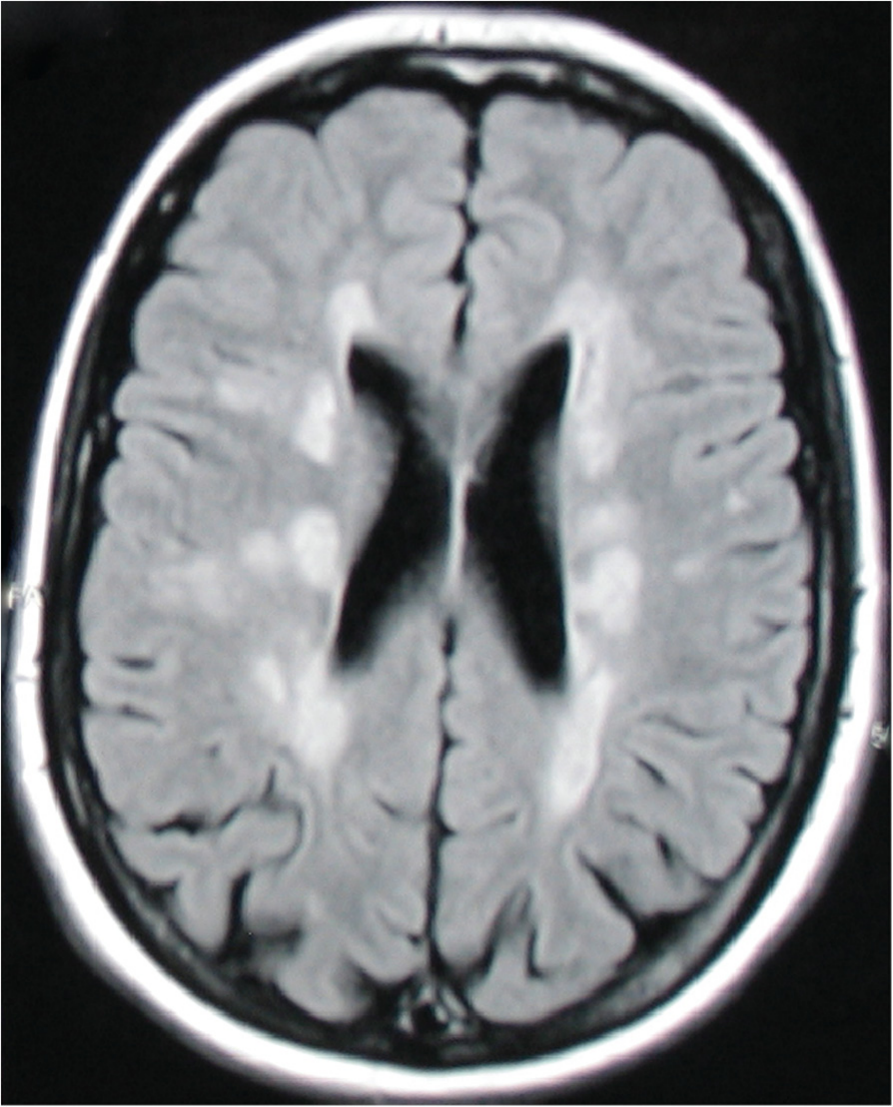
**Brain MRI abnormalities in an anti-MOG antibody-positive patient.** Axial T2-FLAIR (fluid-attenuated inversion recovery) brain MRI of a MOG-seropositive NMO patient 31 years after disease manifestation with combined optic neuritis and transverse myelitis showed multiple abnormalities fulfilling Barkhof criteria and resembling multiple sclerosis lesions.

MOG-seropositive patients showed brain lesions during the disease course more frequently (2/4) as compared to AQP4-seropositive (5/31) and seronegative (1/13) patients. Follow-up brain MRI in two MOG-seropositive patients revealed juxtaventricular lesions in one patient, and lesions in the thalamus and corpus callosum in another patient fulfilling the Barkhof criteria (Figure 2). All of the patients had longitudinally extensive transverse myelitis (LETM) in the cervical and/or thoracic spinal cord.

**Figure 3:**
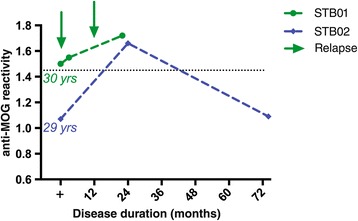
**Longitudinal follow-up of MOG-seropositive patients of up to 6 years reveals fluctuating anti-MOG antibody levels.** Longitudinal serum samples were available for two of the four anti-MOG antibody-positive patients. Serum samples were taken 30 or 29 years after disease onset over a time course of about 2 or 6 years (22 or 74 months). Anti-MOG reactivity is expressed as the geometric mean channel fluorescence (GMCF) ratio. The cutoff used (dotted line) is the mean GMCF ratio of the healthy donor group measured in parallel (*n* = 39) plus two standard deviations (cutoff = 1.45). The arrow indicates a clinical relapse. Antibodies against MOG fluctuated over the disease course: An increase of anti-MOG antibodies was associated in one patient (STB01) with an increased relapse frequency, but was independent of disease activity in the other patient (STB02).

Longitudinal follow-up samples, which were available in two patients with anti-MOG antibodies, showed fluctuating antibody levels over the follow-up period of 22 and 74 months (Figure 3). An increase of anti-MOG antibodies was associated in one patient (STB01) with an increased relapse frequency, but was independent of any disease activity in another patient (STB02) (Figure 3).

## Discussion

We identified four patients with antibodies against native conformational MOG in a blinded cohort of 48 NMO/NMOSD patients. All four patients fulfilled the diagnostic criteria of NMO at disease onset (1/4) or at the time of second territorial involvement (3/4). Anti-MOG antibodies were detected in 4 out of 48 NMO/NMOSD patients and 4 out of 17 AQP4-seronegative patients. These findings are in line with other recent reports that identified antibodies against MOG in a subgroup of AQP4-seronegative patients with NMO/NMOSD [8-10,12-16] (Table 2).

**Table 2:** **Review of literature on anti-MOG antibodies in adult NMO/NMOSD**

**Publication**	**MOG+ NMO (SD) (%AQP-seroneg.)**	**Assay**	**Characteristics of MOG+ NMOSD patients**		
			**Gender (%fem.)**	**Clinical presentation/MRI**	**Outcome (median f/u in months)**
Mader [8]	*n* = 10	CBA	70	n.a.	n.a.
	(39%)				
Kezuka [9]	*n* = 8	ELISA	n.a.	n.a.	No improvement of visual acuity
	(57%)				
Kitley [10]	*n* = 4	CBA	25	Monophasic simultaneous or sequential TM and ON; lower spinal cord involvement on MRI	Excellent recovery (12)
	(15%)				
Sato [13]	*n* = 16	CBA	38	More restricted phenotype (ON > TM); bilateral simultaneous ON; single attack; lower spinal cord involvement on MRI	Good recovery in 88% (24)
	(21%)				
Kitley [12]	*n* = 9	CBA	44	Simultaneous/sequential ON/TM; conus and deep gray nuclei involvement on MRI	Better outcomes; less risk for disability (18)
	(35%)				
Höftberger [16]	*n* = 16	CBA	53	Higher frequency of involvement of all spinal cord regions	Better outcomes; less risk for disability (67)
	(16%)				
Ramanathan [14]	*n* = 9	CBA	67	Strong association with BON; optic disk swelling	Propensity to relapse (28)
	(39%)				

Summary of the published literature on anti-MOG antibodies in adult NMO/NMOSD listed in PubMed until October 2014: The first three publications of the table summarize the literature not solely focused on NMO/NMOSD, while the other reports summarize the publications with a systematic analysis of anti-MOG antibodies in NMO/NMOSD. Abbreviations: BON, bilateral optic neuritis; CBA, cell-based assay; ELISA, enzyme-linked immunosorbent assay; fem., female; f/u, follow-up; MOG, myelin oligodendrocyte glycoprotein; n.a., not available; NMO, neuromyelitis optica; NMOSD, neuromyelitis optica spectrum disease; ON, optic neuritis; TM, transverse myelitis.

MOG-seropositive patients tended to have an earlier or even pediatric onset in our cohort. Two recent studies already indicated that anti-MOG antibodies are present in some pediatric NMO patients [15,17]. Since our samples were taken in adulthood later during the disease course, one can speculate that anti-MOG antibodies might develop early on and persist over many years in some of these patients. Since anti-MOG antibodies are also found in pediatric MS and acute disseminated encephalomyelitis (ADEM) [18,21-26] the picture emerges that the age of disease onset influences the nature of the autoantigen.

Furthermore, compared to AQP4-seropositive and to seronegative patients, the anti-MOG-positive patients in our group resembled a more MS-like phenotype with more common brain involvement during the disease course and positive OCBs in the CSF. The presence of OCBs is different from other recent reports which have found no or less frequent OCBs in the MOG-seropositive NMOSD patients [12-16]. Regarding brain lesions on MRI in the MOG-seropositive group, the lesions were more reminiscent of MS than NMO lesions with supratentorial, periventricular localization. Moreover, brainstem lesions, which are a hallmark of AQP4-seropositive NMO, were absent in our MOG-seropositive patients, which has also been suggested by other recent reports [13,15].

Our long-term follow-up enabled us to also analyze the disease course over up to 44 years. Looking at the temporal dynamics of anti-MOG antibodies in two patients over up to 6 years indicates a fluctuating pattern of these antibodies as it has been seen in pediatric MS and NMO cases [17,18,27]. It is therefore necessary to test patients longitudinally to assess anti-MOG serostatus.

### Conclusion

We identified anti-MOG antibodies in a subgroup of anti-AQP4 antibody-negative NMO patients (about 25%), but not in anti-AQP4 antibody-positive patients. Interestingly, some of the MOG-seropositive patients presented with a pediatric disease onset. The MOG-seropositive patients might show a more benign clinical course with a lower relapse rate and a longer time to a second attack affecting a different CNS region compared to the AQP4-seropositive and seronegative patients.

Our data suggest that MOG-seropositive patients show a diverse clinical phenotype with clinical features resembling NMO (attacks confined to the spinal cord and the optic nerves) and MS with an opticospinal presentation (positive OCBs, brain lesions). Further studies with larger cohorts need to be conducted to consolidate these findings and potentially lead to therapeutic recommendations which also address the seemingly more benign clinical course with a lower relapse frequency in the majority of the MOG-seropositive patients.

## Abbreviations

ADEM: acute disseminated encephalomyelitis
AQP4: aquaporin-4
CBA: cell-based assay
CNS: central nervous system
FLAIR: fluid-attenuated inversion recovery
GMCF: geometric mean channel fluorescence
LETM: longitudinally extensive transverse myelitis
MOG: myelin oligodendrocyte glycoprotein
MRI: magnetic resonance imaging
NMO: neuromyelitis optica
NMOSD: neuromyelitis optica spectrum disorder
OCBs: oligoclonal bands
ON: optic neuritis
RRMS: relapsing-remitting multiple sclerosis
TM: transverse myelitis
